# The “second empty nest”: The lived experiences of older women whose intensive ‘grandmotherhood’ ended

**DOI:** 10.1093/geroni/igaf122.1897

**Published:** 2025-12-31

**Authors:** Yarin Cohen, Gabriela Spector-Mersel, Sharon Shiovitz-Ezra

**Affiliations:** University of South Carolina, Columbia, South Carolina, United States; Sapir College, Sderot, HaDarom, Israel; The Hebrew University of Jerusalem, Jerusalem, Yerushalayim, Israel

## Abstract

In Israel, grandmothers constitute the primary source of informal childcare. This intensive caregiving refills “the empty nest” and aligns with core Israeli cultural values: it reinforces the active Sabra ethos (contrasting with the stereotype of the Diaspora Jew as passive), and maintains the grandmother`s feminine identity through childcare—central to womanhood in Israel’s pro-natalist society. This study examined the lived experiences of Israeli older women whose intensive grandmotherhood has diminished or ceased. Through in-depth interviews with 11 grandmothers and Moustakas’ three-phase phenomenological analysis, we identified three key themes: a view of the active grandmother role as a second motherhood, the difficulties experienced following cessation of intensive caregiving, and the strategies employed to cope with the resulting void. Participants’ experiences showed both positive and negative aspects of the intensive grandmotherhood. After cessation of their caregiving role, some showed remarkable similarities to mid-life mothers experiencing the “empty nest” syndrome. However, the “second empty nest” seems to pose more profound identity challenges, as it represents a final passage into what is culturally constructed as passive, genderless old age. Accordingly, grandmothers are required to employ various strategies to address these challenges. The “second empty nest” pertains to the experience of older women in many countries, but seems particularly intense within Israeli society, given the cultural emphasis on activity as characteristic of “worthy” (non-old) individuals and childcare as a defining element of (non-old) femininity. These findings demonstrate how deeply personal experiences of older adults are rooted in the socio-cultural construction of old age.

